# Initiation of a stable convective hydroclimatic regime in Central America circa 9000 years BP

**DOI:** 10.1038/s41467-020-14490-y

**Published:** 2020-02-05

**Authors:** Amos Winter, Davide Zanchettin, Matthew Lachniet, Rolf Vieten, Francesco S. R. Pausata, Fredrik Charpentier Ljungqvist, Hai Cheng, R. Lawrence Edwards, Thomas Miller, Sara Rubinetti, Angelo Rubino, Carla Taricco

**Affiliations:** 1 Department of Earth and Environmental Systems, Indiana State University, Terre Haute, 47809 IN USA; 2 Department of Marine Sciences, University of Puerto Rico, Mayaguez, 00680 Puerto Rico USA; 3 University Ca’ Foscari of Venice, Department of Environmental Sciences, Informatics and Statistics, Via Torino 155, 30172 Mestre, Italy; 4 Department of Geoscience, University of Nevada Las Vegas, Las Vegas, 89154 NV USA; 5 Institute of Earth Sciences, Hebrew University of Jerusalem, Jerusalem, Israel; 6 Centres ESCER (Étude et la Simulation du Climat à l’Échelle RÉgionale) and GEOTOP (Research Center on the dynamics of the Earth System), Department of Earth and Atmospheric Sciences, University of Quebec in Montreal, Montreal, QC Canada; 7 Department of History, Stockholm University, SE-106 91 Stockholm, Sweden; 8 Bolin Centre for Climate Research, Stockholm University, SE-106 91 Stockholm, Sweden; 9 Department of Earth Sciences, University of Minnesota, Minneapolis, MN 55455 USA; 10 School of Human Settlement and Civil Engineering, Xi’an Jiaotong University, Xi’an, 710049 China; 11 Department of Geology, University of Puerto Rico, Mayaguez, 00680 Puerto Rico USA; 12 Physics Department, University of Torino, via Pietro Giuria 1, 10125 Torino, Italy; 13 Istituto Nazionale di Astrofsica, Osservatorio Astrofisico di Torino (OATo-INAF), Strada Osservatorio 20, 10025 Pino Torinese, Italy

**Keywords:** Climate sciences, Palaeoclimate, Ocean sciences

## Abstract

Many Holocene hydroclimate records show rainfall changes that vary with local orbital insolation. However, some tropical regions display rainfall evolution that differs from gradual precessional pacing, suggesting that direct rainfall forcing effects were predominantly driven by sea-surface temperature thresholds or inter-ocean temperature gradients. Here we present a 12,000 yr continuous U/Th-dated precipitation record from a Guatemalan speleothem showing that Central American rainfall increased within a 2000 yr period from a persistently dry state to an active convective regime at 9000 yr BP and has remained strong thereafter. Our data suggest that the Holocene evolution of Central American rainfall was driven by exceeding a temperature threshold in the nearby tropical oceans. The sensitivity of this region to slow changes in radiative forcing is thus strongly mediated by internal dynamics acting on much faster time scales.

## Abstract (web summary)

What drives hydroclimate changes in tropical regions is not well known. Here, the authors present a 12,000 year long precipitation record from Guetemala which shows that exceeding a threshold in sea surface temperatures caused Central American rainfall to change from a dry to an active convective regime around 9000 years ago.

## Introduction

On orbital time scales, many paleoclimate records in tropical regions are linked to gradual variations in incoming solar radiation^1^ associated with changes in the Earth’s orbital parameters. For example, the Earth’s precession variability produces insolation anomalies on time scales of ~19,000 and ~23,000 years that have led to slow variations in regional climate and hydroclimate over the subtropical latitudes of SouthEast and East Asia, as well as South and North America, as shown by a tight correlation between summer insolation and proxies of integrated precipitation strength^2–7^. In southern Central America, the convergence of moisture related to the Intertropical Convergence Zone (ITCZ) produces heavy convective rainfall over land^2^ with an annual peak between June and October. However, some near-equatorial paleoclimatic records feature characteristics inconsistent with summer insolation changes, e.g., in Australia^3^, on the Pacific coast of Costa Rica in Central America^4^, and in Borneo on the Western Tropical Pacific^5^. Some other records suggest relatively stable Holocene rainfall despite changing insolation, e.g., the Liang Luar Cave record from Indonesia^6^ and the sediment record from Lake Petén Itzá in Guatemala^7^. The contrast between records showing orbital variability and records apparently unaffected by such variability highlights the considerable spatio-temporal complexity of tropical hydroclimates around the globe and their different sensitivity to external forcings.

In Central America the marked heterogeneity of modern hydroclimate regimes arises from interacting atmospheric, oceanic, and land processes that occur in this narrow stretch of land separating the tropical Pacific and tropical North Atlantic oceanic basins^8–12^. The same processes explain the strong sensitivity of this region to external forcing. Stalagmite records suggest that externally forced multidecadal anomalies in Atlantic and Pacific sea-surface temperatures (SSTs) significantly made an imprint on Central American rainfall evolution throughout the past few centuries^8^. Model projections^13^ under global warming scenarios suggest that a similar chain of mechanisms may be responsible for a potential substantial drying of more than 30% over Central America. However, whether such strong sensitivity to warming and associated dynamics is a persistent characteristic of local climate is uncertain due to the few continuous multi-millennial and accurately dated paleoclimate archives for this region^14,15^. Together with knowledge gaps about general long-term hydroclimate variability^16,17^ and difficulties of current climate models to robustly simulate large-scale oceanic and atmospheric phenomena linked to Central American rainfall^18–20^, this uncertainty simply corresponds to a poor understanding of the prime controlling mechanisms of Central American hydroclimate.

Available paleoclimate reconstructions from Mesoamerica do not provide a consistent description of the Central American hydroclimatic state and variability during the Holocene. A speleothem rainfall reconstruction spanning the last 100,000 years from Barra Honda National Park, Costa Rica (10°N; Supplementary Fig. 1) is poorly explained by the orbital paradigm, and it appears instead to be largely controlled by Caribbean-Pacific SST gradients^4^. However, this speleothem record does not capture the end of the last deglaciation and the whole Holocene. In contrast, Mexican speleothems from the Mesoamerican region (18°N; Supplementary Fig. 1) indicate that the local, long-term rainfall evolution during the Holocene was paced by insolation changes^9^. However, the Mexico records also exhibit pronounced millennial-scale variability possibly reflecting a contribution by internal processes, specifically from North Atlantic SSTs and variations in the oceanic thermohaline circulation. Such dynamical interpretations must also account for the possible inconsistencies between hydroclimate reconstructions obtained for the same region from different paleoclimate archives^21^.

**Fig. 1:**
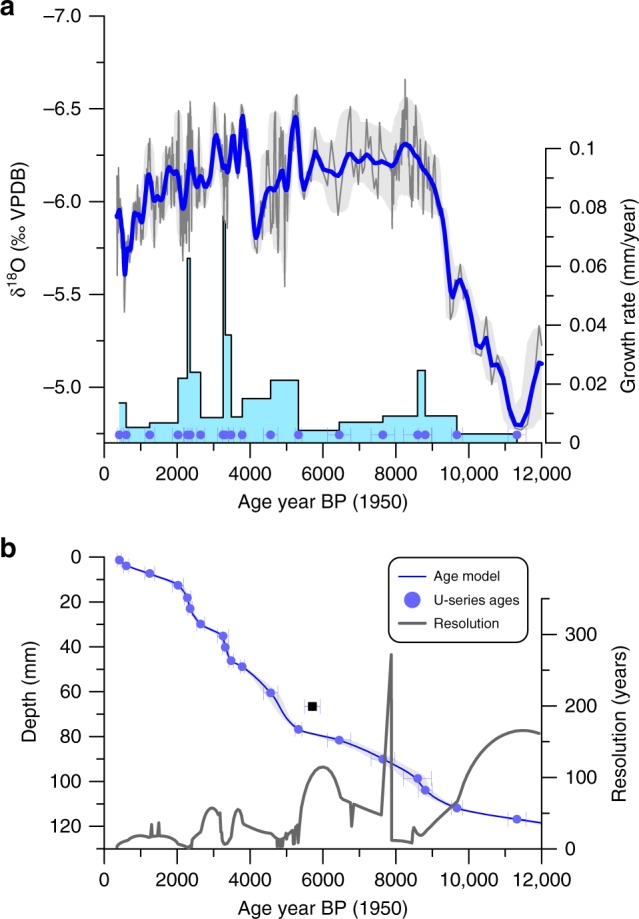
Central American rainfall time series inferred by GU-RM1. **a** GU-RM1 δ^18^O time series (gray line and gray shading: measured values and age uncertainties; bold blue line: COPRA^61^ output time series). **b** Age-depth model for ^230^Th dates with gray uncertainty envelope from the COPRA modeling. The date at 67 mm depth was omitted from the age model and is shown for reference as a black square. The gray line shows the δ^18^O sampling resolution (years between samples).

In this study, we present a new Holocene rainfall-sensitive speleothem record (GU-RM1, Fig. 1, Supplementary Fig. 1; see Methods section for more details) from Rey Marcos cave located at 1460 m altitude (15.4°N, 90.3°W) on the Caribbean slope of Central America in the Guatemalan highlands. This is currently the first and best-dated published high-resolution speleothem record from Central America that continuously spans the Holocene. The data show that variability of Central American rainfall evolved through three major phases: a relatively sharp wetting in the early Holocene (11,000–9000 years before the present (yr BP)), a stable regime from 9000 to 5000 yr BP, a centennial scale drying period between 5000 and 4000 yr BP, and a subsequent tendential drying that persists to the present. Supported by climate model simulations, the transition from a dry into a persistently active convective regime around 9000 yr BP was largely enhanced by tropical Atlantic SST warming, while being relatively insensitive to local insolation variations. Our record represents a significant advance on previous studies because of the high-precision U-series dating of the rainfall strengthening that is unaffected by issues associated with radiocarbon calibration and problems related to carbon cycling in lake sediments.

## Results and discussion

### Wettening in the early Holocene followed by stable conditions

The calcite GU-RM1 speleothem has a robust age model (Fig. 1b) constrained by twenty-one U/Th multi-collector inductively coupled plasma mass spectrometry dates (Supplementary Table 1, Supplementary Fig. 1), which show slow but apparently continuous growth from ~12,700 yr BP (below the lowest date at 11,320 ± 247 yr BP) to ~300 yr BP (see Supplementary Information). This slow growth preserves a uniquely long Holocene climate history for this humid tropical region. Because the amount effect is the dominant control on δ^18^O variations in Central America^22^ and the Yucatán lowlands^23^, and the Caribbean slope is dominated by a single oceanic moisture source (the Caribbean Sea), we interpret the δ^18^O variations in the stalagmite as reflecting regional convective intensity. The most prominent aspects of our record are a sharp decrease of 1.3‰ in δ^18^O values in the early Holocene (between 11,000 and 9000 yr BP), linked to increased rainfall over Central America, and the following period of relatively stable conditions during the last 9000 yr BP, when δ^18^O values range between −6.6 and −5.5‰ VPDB (Fig. 1a). Our reconstruction contains a significant but small drying trend of 0.0325 ± 0.0037‰/1000 yr over the last 9000 yr BP. The last 3.5 millennia exhibit a trend from −6.5 to −5.5‰ VPDB indicative of reduced precipitation as commonly reported for other records^1,9,10,15^.

**Fig. 2:**
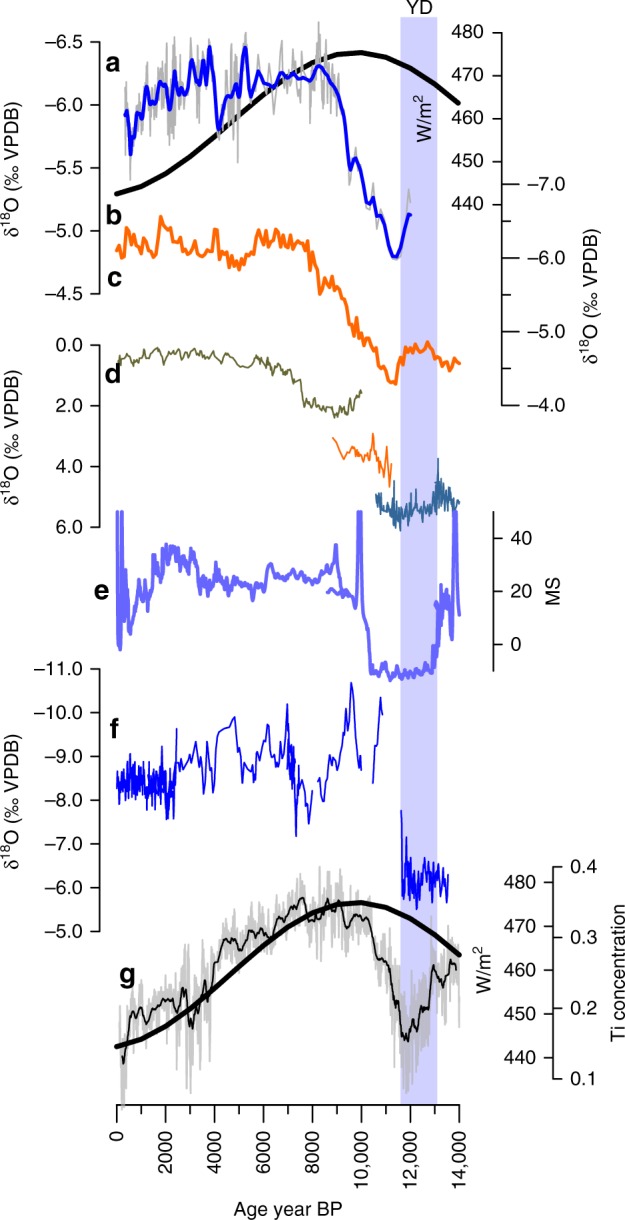
Comparison of Central American and regional rainfall records. **a** Guatemala (GU-RM1) δ^18^O (proxy for precipitation). **b** 15°N insolation for Jul 21. **c** Lake Petén Itza δ^18^O (proxy for precipitation) from three different locations in the basin^26,67,68^. **d** The δ^18^O (proxy for precipitation) record from Juxtlahuaca and Diablo Caves^9^, Mexico. **e** Petén Itza magnetic susceptibility (environmental finger printing)^7,26^. **f** Juxtlahuaca cave, Mexico^69^. **g** Cariaco Basin titanium concentration (hydrological input) record 28 and July 21 insolation at 15°N.

The main characteristics of the Guatemalan rainfall record from GU-RM1 agree with the characteristics found in the Petén Itzá lacustrine sediment δ^18^O record^24^, which describes changes in the balance between precipitation and evaporation in the Guatemalan lowlands (Fig. 2a, d) and in the Lake Quexil pollen record^25^. Specifically, all records depict the dry-to-wet transition between 11,000 and 9000 yr BP with the relatively stable hydroclimate conditions thereafter, and a drying trend is established during the last two millennia. The agreement between the GU-RM1 record of precipitation intensity and the Petén Itzá δ^18^O record of precipitation minus evaporation suggests that increased rainfall amount is also related to a decrease in evaporation as evident in lower δ^18^O values in the lake record. Because the GU-RM1 record is precisely dated and is less influenced by evaporative increases in δ^18^O like Lake Petén Itzá, it is a more direct proxy of variations in the δ^18^O of precipitation. The beginning of the dry-to-wet transition has a similar onset in both the Cariaco Basin^26,27^ (Fig. 2g) at ~11,600 yr BP and GU-RM1 at ~11,400 yr BP, well within the age uncertainty of our age model at this time (±260 years). While the transition to early Holocene wetness in the Cariaco record was mostly complete by ~10,300 yr BP and coincident with the peak in July 21 insolation, the transition in Guatemala did not reach completion until ~9000 yr BP. Rainfall then decreases abruptly at ~4000 yr BP in northern South America, while Guatemala rainfall decreased gradually in the Late Holocene starting 3800 to ~400 yr BP. These contrasting observations reveal a latitudinal differentiation of ITCZ-related rainfall.

Interestingly, the GU-RM1 δ^18^O record is not correlated with July 21 insolation during the last 12,000 yr BP (*r*(*p*) = −0.049 (0.925), see also Fig. 2a, b). Indeed, the shape of the GU-RM1 curve does not exhibit a sine-wave like evolution over the course of the Holocene, as would be predicted if orbital insolation in any month was the dominant control on Central American rainfall. This contrasts with other nearby rainfall hydroclimate records, including the Cariaco Basin which closely follows July 21 insolation, at essentially the same latitude of 15°N. The correspondence of boreal summer insolation and the latitudinal migration of the ITCZ has underpinned the dominant paradigm of climatic variation on Holocene time scales for the neo-tropics, yet it fails to explain the full Holocene evolution of rainfall in Guatemala. Further, local summer insolation has been used to interpret the Huagapo speleothem δ^18^O record^28^ (not shown) from South America, and the Juxtlahuaca and Diablo Caves (Mexico) which respond to a mix of orbital and ocean circulation pacing (Fig. 2d). Similarly, the monsoon over southwestern Mexico^9^ appears to have strengthened by ca. 11,000 yr BP after the aridity of the Younger Dryas (Fig. 2f), possibly linked to the resumption of the Atlantic Meridional Overturning Circulation (AMOC), suggesting that the forcings there differ from those in Guatemala.

The GU-RM1 record spanning the whole Holocene provides a new perspective on Central American hydroclimate evolution and confirms that Central America transitioned into a wet regime similar to the modern one no earlier than 9000 yr BP, following by more than a millennium the cessation of gypsum deposition indicating arid conditions in Lake Petén Itzá at around 10,300 BP^29^. The wet regime has persisted ever since, while at the same time undergoing multicentennial to millennial-scale variations. The different reconstructed rainfall evolution highlights the marked hydroclimatic heterogeneity of the Central American region.

### Evidence of deglaciation forcing and oceanic circulation

**Fig. 3:**
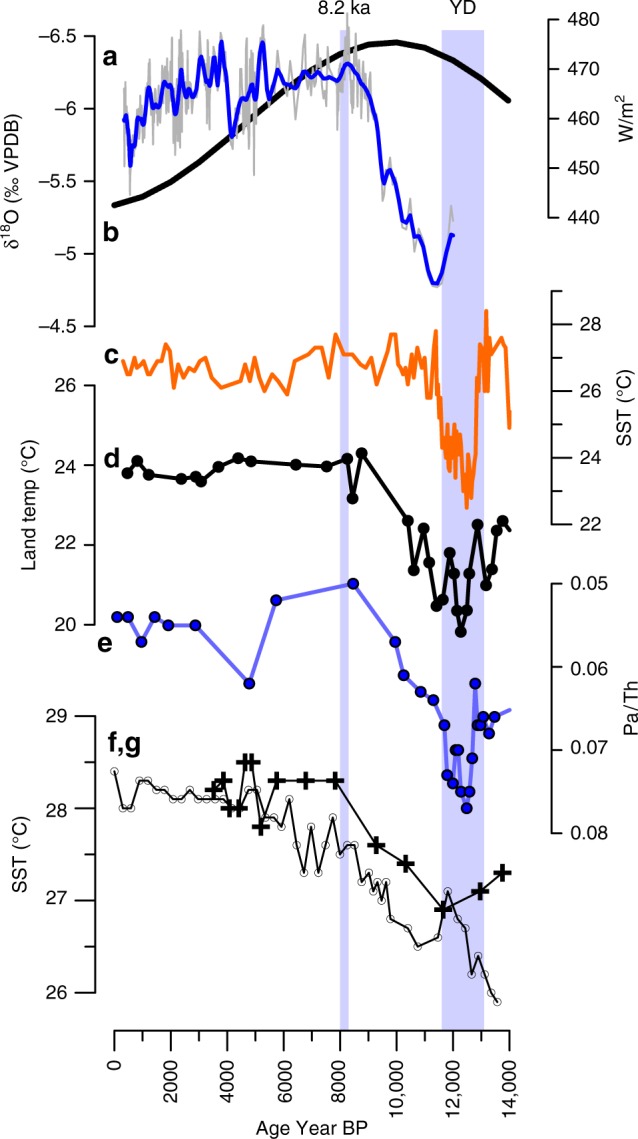
Ocean–atmosphere linkages to the Holocene rainfall increase. Comparison of **a** the Guatemala speleothem GU-RM1 precipitation proxy with **b** 15°N July 21 insolation, **c** sea-surface temperature (SST) in the Cariaco Basin from Mg/Ca proxy^57^, **d** the inferred land surface temperature for the Petén region of Guatemala from pollen in Lake Petén Itza sediments^7^, and **e** the Pa/Th ratio^70^, a proxy for the strength of the Atlantic Meridional Overturning Circulation (AMOC), **f** the SST near Grenada^33^ (gray line with circles), and **g** Caribbean SST^32^ (black line with crosses).

The transition from a dry regime to the early Holocene wet regime in the GU-RM1 record between ~11,000 and 9000 yr BP follows the late phase of quickly retreating ice sheets. This period was associated with a strengthening of the AMOC, increase in Caribbean SSTs^30,31^, and warming land surface temperatures^7,32^ (Fig. 3). Indeed, the time of peak AMOC strength at 8400 yr PB changes synchronously (within age model uncertainties) of peak rainfall amount at 8400 yr BP as inferred from the lowest GU-RM1 δ^18^O values. Further, the Caribbean SST warming is part of a larger surface ocean warming in the tropical North Atlantic as shown by SST reconstructions from the eastern part of the basin (Fig. 3f) and by indications of early post-glacial warming in reconstructions of Gulf of Mexico SSTs^33,34^.

We thus posit that Caribbean SST and land surface warming was manifested as the basin-scale response to a more vigorous AMOC. Variations in the strength of the AMOC are linked to meridional redistributions of ocean heat within the North Atlantic whose SST spatial pattern superposes on that of the Atlantic Multidecadal Oscillation (AMO)^35,36^. The AMO is a mode of natural climate variability that consists of the multidecadal alternation between warm and cold phases of North Atlantic average SST anomalies^37^, which are known to entail surface warming and cooling of the inter-American Seas^38^. Reconstructions of multidecadal climate oscillations suggest that AMO-like variability existed during large parts of the Holocene^39^. A tight connection between AMO variability and Central American rainfall has been shown to hold during the last ~2.5 centuries^8^ and seems to be active as well on longer (millennial and longer) time scales^40^. The nature of the AMO as a predominantly oceanic rather than atmospherically forced phenomenon remains debated^41–44^. Notwithstanding uncertainties in the AMO nature and characteristic time scales, we build on the AMO paradigm to better understand the possible near-surface dynamics contributing to basin-average North Atlantic SST changes and Caribbean hydroclimate variations in the early Holocene, i.e., we do not claim that AMO necessarily explains—but is a useful analog for—the early Holocene regime shift for Caribbean rainfalls.

**Fig. 4:**
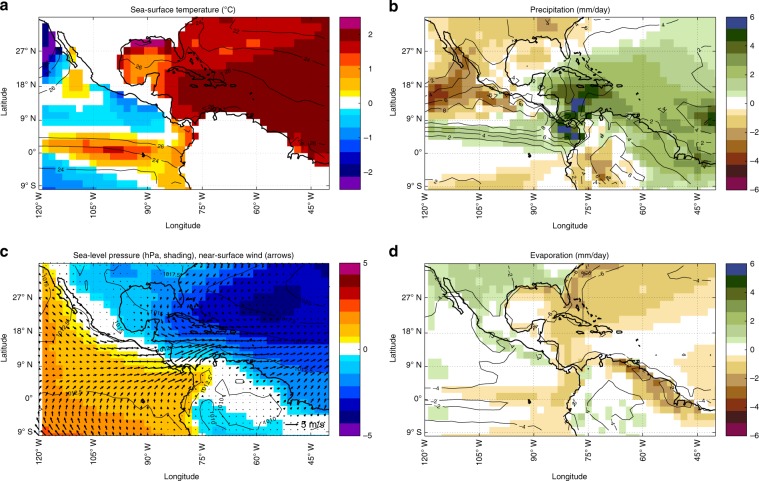
Climate anomalies with a ~2 °C tropical Atlantic SST warming. Idealized simulation with the Max Plank Institution for Meteorology Earth System Model. Shadings are anomalies in sea-surface temperature (**a**), precipitation (**b**), sea-level pressure (**c**), and evaporation (**d**) between peak warm Atlantic Multidecadal Oscillation (AMO) and peak cold AMO anomalies from six idealized 70-year sinusoidal AMO cycles; line contours identify the associated climatologies (see ref. ^45^ for details). Arrows in panel **c** represent warm-cold differences in 10 m winds. Peak warm and peak cold values are determined as 11-year averages around the warmest and coldest year in each AMO cycle, respectively.

Accordingly, idealized AMO climate simulations^45^ yielding peak warm-minus-cold differences in Caribbean SSTs comparable to reconstructed estimates of post-glacial warming result in a broad precipitation increase over the Caribbean region. This is particularly linked to the development of a near-surface negative pressure anomaly in the western tropical Atlantic that weakens the climatological flow as evidenced by the westerly wind anomalies in the Caribbean low-level jet core region (Fig. 4). Such interpretation is consistent with wetting over the Yucatan based on arguments about moisture divergence^46,47^. Other climate simulations further show that changes in the extent of Northern Hemisphere’s ice sheets are associated with changes in inter-hemispheric temperature gradients, in turn driving meridional ITCZ shifts through modified tropical-midlatitude atmospheric bridges and associated heat transport^48^. The reconstructed change in the Central American precipitation regime is linked to warming of Caribbean SSTs as part of a basin-scale, AMOC-driven warming of the upper North Atlantic with a spatial pattern like the one characterizing modern multidecadal SST variability. We thus attribute increased rainfall amounts to surface ocean warming in the Caribbean Sea, which is the dominant moisture source feeding rainfall at the GU-RM1 site, associated with the invigoration of AMOC following the decay of the Laurentide Ice Sheet. Aspects of the mechanisms linking AMOC, SST, and Caribbean rainfall remain to be understood, particularly regarding the quantifications of time lags.

### Regional dynamic interpretation

With the culmination of the Laurentide ice sheet melting around 8000 yr BP, SST warming in the tropical North Atlantic contributed to further northward extension of the area under the influence of the ITCZ. The SST warming and the ITCZ shift led to increasingly stronger atmospheric convective activity over Central America, until a critical threshold was reached to persistently include Guatemala at around 9000 yr BP. In contrast to regions within the core of the ITCZ, such as the Cariaco basin (10°N), which predominantly experienced changes in rainfall intensity, areas at the edge of the ITCZ experienced a transition from a predominantly non-convective to a predominantly convective regime. This is an important difference since the rainfall proxy record from Cariaco cannot be seen as representative of the broad rainfall evolution over Central America. Our data also provide long-term context to the hydroclimatic variations during the late Holocene that may have impacted ancient indigenous Mesoamerican societies such as the Olmecs and the Maya^49^: such variations occurred within the general stability of the hydroclimatic wet regime and are much smaller than the dry-to-wet transition during the early Holocene. However, relatively small variations in rainfall have been shown to impact past societal responses to climate change^50^, suggesting that future rainfall change within the active convective regime may also have significant societal effects^51^.

Within a simplified dynamical framework, the mean value of reconstructed Caribbean SST after the early Holocene transition remains around the estimated critical SST threshold of ~27.5 °C needed to trigger atmospheric deep convection in the tropics^52^. However, the covariation between tropical-mean SSTs and the convective threshold revealed by satellite data^53^ suggests that such a threshold depends, to some extent, on the background climate conditions, and it might have been lower than for present-day climates in the early post-glacial era. A faunal record of SST variations off West Africa^54^ reveals a major warming of ~2 °C around 5000 yr BP, which might have implications for the evolution of Central American precipitation, as the GU-RM1 and Cariaco records diverge most noticeably following such transition.

We conclude that precipitation on the Caribbean slope of Central America was not predominantly affected by insolation but by a change in the convective regime associated with the disappearance of the Laurentide Ice Sheet and strengthening of the AMOC, leading to a broad warming in the tropical North Atlantic and ultimately a broadening of the area under the ITCZ influence. In glacial times, relatively cold Caribbean SSTs (as low as 23 °C, see Fig. 3g) could not sustain vigorous convective activity in the Guatemalan region, whereas SST warming during the glacial–interglacial transition and persistent warm SST conditions through the Holocene favored increased convective activity there, and a decrease in rainfall δ^18^O values. Reconstructed SSTs in the Cariaco basin underwent a sharp warming to reach ~ 27 °C around 10,000 years^55^, i.e., before the onset of an active hydrological regime in our record and maximum rainfall-induced runoff to the Cariaco Basin at ~9000 yr BP (Fig. 3c). Quantification of the lag between both events requires narrowing and more robustly constraining age model uncertainties.

In summary, the Guatemalan GU-RM1 speleothem record reveals crucial information about the evolution of Central American rainfall throughout the Holocene and sheds light on dominant dynamics underlying hydroclimate variability in the area, particularly concerning its sensitivity to external forcing. Our study shows that Central American rainfall as inferred by the Guatemalan GU-RM1 speleothem record underwent an “off-on” switch between 11,000 and 9000 yr BP. This increase in precipitation at the GU-RM1 site was likely enhanced by AMOC recovery, which contributed to SST warming in the Caribbean Sea and northward shift of the ITCZ, bringing the pool of atmospheric moisture in closer proximity to Guatemala, to which it supplied convective systems over land. The exceedance of an SST threshold in the nearby tropical ocean triggered deep atmospheric convection, hence a dry-to-wet regime shift over Central America. Paleoclimate records from the Cariaco basin indicate that weakening of summer insolation during the middle and late Holocene led to a gradual southward retreat of the ITCZ. However, our results suggest that the Central American rainfall did not exhibit a large decrease directly following changes in the insolation forcing but remained strong due to the convective activity favored by persisting warm SSTs in the Caribbean Sea. Further, our results support recent model-based evidence of a significant role of the AMOC in the modulation of externally forced latitudinal ITCZ shifts^56^.

From this speleothem record the Central American hydroclimate emerges as strongly linked to SST variability in the Caribbean and tropical North Atlantic. The association between Central American rainfall variability and basin-scale oceanic changes in the North Atlantic seen on multidecadal-to-centennial time scales for the past 1200 years^36^ thus appears to hold also on Holocene time scales. Our work is also intended to stimulate further research to characterize and understand hydroclimate variability in a narrow land bridge of strong topographic heterogeneity at the boundary between two major ocean basins. These differences stress the need to surpass the simplistic perception of tropical hydroclimates being summarized as a stronger global monsoon in the early- to mid-Holocene compared to the present. Finally, as our results highlight, the dependency of the Central American rainfall on the overturning circulation in the Atlantic Ocean will crucially affect, together with atmospheric circulation changes^47,57^, the future Central American hydroclimate regime.

## Methods

### Stalagmite specimen

The stalagmite GU-RM1 was collected on 8 August 2011 at Grutas del Rey Marcos (Supplementary Fig. 1), located about 20 km southeast of the city of Cobán, Guatemala. Handheld GPS gives a location of 15.42769°N and −90.28066°W, at an elevation of 1460 m asl. The area is temperate and damp year-round, with a high-altitude tropical forest covering the region of the cave, with a mean annual precipitation of 2300 mm and a temperature of 18 °C. Mean monthly relative humidity is above 80% in the wet season, dropping to ~75% during the March–April dry season. Stalagmite GU-RM1 was recovered 50 m inward from the cave entrance past a narrow constriction in the first main passage. Although the surface of the stalagmite was damp, indicating 100% relative humidity and later verified by in-cave measurements in March 2019, the drip rate at the stalagmite location was too slow at time of sampling to collect drip water samples, or to verify the sample was actively growing; the U/Th data suggest a tip age from 1.3 mm depth of 411 yr BP, possibly indicating inactive growth during collection because we would expect an age closer to 100 yr BP. Rey Marcos cave contains other calcite stalagmites up to several meters in height, which could provide evidence of hydrological changes over several glacial–interglacial cycles. Ongoing cave monitoring was recently initiated in March 2019 to provide cave climate, drip rate, and drip water δ^18^O values; these data will be included in future work.

Stalagmite GU-RM1 has a depressed drip dish flanked by a raised rim similar to many stalagmites we have worked with (Supplementary Fig. 2); the rim is likely formed when the drip impact causes a splash away from the impact point. The milling transect was oriented vertically along the right-hand rim to maintain sample continuity and to avoid calcite in the drip dish, which has less visible banding than along the rim. The precise center of the growth axis moved off the milling axis for some portions of growth. Our experience with other similar stalagmites in humid tropical caves is that isotope values are relatively constant along layers near the growth axis, so that such changes in location are unlikely to have a significant influence on the measured δ^18^O and δ^13^C values. Evaluation of Supplementary Fig. 2 shows that there is no clear isotopic anomaly associated with where our milling transect departs from the central growth axis (~45–90 mm depth), indicating that the isotope data are not influenced by drilling location. However, to test this idea more rigorously, we repeated drilling transects to more closely follow the center of the growth axis (to the side of the drip dish). Supplementary Figure 3 shows that transect location relative to the central growth axis results in the same decrease in from the two different transects meet the null hypothesis that they come from a population with the same mean, as determined by a two-sample *t*-test.

### Age model

An amount of ~200 mg of powder was collected with a handheld dental drill from a polished central slab section of GU-RM1 along growth layers for MC-ICPMS ^230^Th/U-dating. Twenty-one ^230^Th/U dates were analyzed in the upper 117 mm resulting in an age control point approximately every 600 years (Fig. 1b). Sample depths were determined by digitizing a high-resolution image to provide precise mid-point depths. Dates were obtained with a magnetic sector multi-collector inductively coupled plasma mass spectrometer at the Universities of Minnesota and Xi’an Jiaotong (China), following routine instrumental procedures and^58,59^ chemical separation^60^.

U-series ages for stalagmite GU-RM1 are in correct stratigraphic order (except for around 67 and 77 mm depth) and show precise U–Th dates (median two sigma age uncertainty is ±132 years). The high dating precision arises from the high δ^234^U values (>450‰) and a minor contribution of detrital ^230^Th, resulting in small age corrections (<70 years). The robust age/depth progression suggests little to no secondary alteration or post-depositional mobilization of uranium. Two dates near the base are identical within age error (113.6 and 116.8 mm, Supplementary Table 1), so we have elected to use in the age model only the 116.8 mm age (11,320 ± 247 yr BP) because it has the lower uncertainty. Additionally, one section shows an age inversion, of either the 67 or 77 mm samples. We chose to exclude the sample at 67 mm depth from the age model, although it remains plotted in Fig. 1, because it had larger age uncertainty (5710 ± 214 yr BP) compared to the more precise age at 77 mm depth (5328 ± 91 yr BP). The age constraints over the ~65–80 mm interval are thus potentially weaker than at other sections of the stalagmite; a sensitivity test to swap in the 67 mm age and swap out the 77 mm age in the modeling routine suggests a difference in ages of <500 years for this depth interval compared to the age model used in this paper, although it does not affect the overall conclusion of the paper. Future replication with other stalagmites may resolve the discrepancy.

The age model for the speleothem is calculated using the COPRA algorithm^61^ based on the ^230^Th/U dates and distance from the top using a Monte Carlo simulation of 2000 possible age models. The age model uncertainty (Supplementary Fig. 2) was estimated from the COPRA Monte Carlo age/depth simulations and is mostly better than ±400 years. We show in each plot (Figs. 1–3, panel a) both the COPRA output time series (bold blue lines) that accounts for age model uncertainty, and the age assignment to each measured δ^18^O subsample (thin blue line). We focus our discussion on the post-Younger Dryas interval, because our age model is not robust for the oldest sections of the stalagmite.

Finally, the GU-RM1 stalagmite was slow-growing (0.012 mm/yr over the last ~11,000 years). Such slow growth in warm humid tropical caves is unusual and provide a unique opportunity to constrain a Holocene-length climate record as many other stalagmites from the humid tropics have growth rates one to two orders of magnitude faster than GU-RM1 and span shorter time intervals. We consider that this specific stalagmite was possibly beneath a slow but continuous drip, which allowed growth much slower than typically seen for such wet climates. We will further test this idea by the collection of additional material from the cave in future visits.

### Local climatology

The central Guatemala highlands currently features a hydroclimate regime characterized by a boreal summer/fall rainy season and a relatively dry winter^8,62^. This annual rainfall cycle is largely determined by the seasonal migration of the ITCZ, whose northernmost position in boreal summer currently reaches Mesoamerica (Supplementary Fig. 5), by the summer intensification of the easterly Caribbean low-level jet determining an increased moisture transport and precipitation over Central America, and by topographic effects. The stalagmite most likely reflects wet season recharge, as suggested by drip water observations in a Yucatán lowland cave ^23^.

### Isotopic analysis

A Sherline 5410 mill was used to continuously mill powder samples near the growth axis for stable isotope analysis at a resolution of 0.1–0.5 mm (Supplementary Fig. 6). The subsampling intervals result in a median sample resolution of 12 years, with the majority (83%) of the samples having an age resolution better than 40 years, and 69% of the samples having a resolution better than 20 years based on the COPRA age model output (Fig. 1). Stalagmite δ^18^O values were determined on a Kiel IV automated carbonate preparation device via phosphoric acid digestion at 70 °C connected to a ThermoElectron Delta V Plus mass spectrometer. Samples were corrected to an in-house calcite standard that was calibrated to NBS-19 and NBS-18 standards and reported relative to the VPDB (Vienna PeeDee Belemnite) international standard. Precision on δ^18^O is better than ±0.08‰ and for δ^13^C is ±0.06‰. The analysis was conducted at the Las Vegas Isotope Science Laboratory at the University of Nevada Las Vegas. The lack of δ^18^O/δ^13^C covariation (Supplementary Fig. 4) over the Holocene indicates that kinetic fractionation is not a strong control on stable isotope values. To further test this idea, we estimated the drip water δ^18^O value using the cave temperature measured on 22 and 23 March 2019 of 17.6 °C and our uppermost calcite sample with a δ^18^O value of −6.1‰ VPDB. This sample likely does not represent modern calcite precipitation, because the top U-series age at 1.3 mm returned an age of 411 ± 57 yr BP but is used because it is the most recent available. The corresponding equilibrium δ^18^O value of the drip is −7.0‰ VSMOW using the most up to date calcite-water fractionation equation^63^. These data compare to δ^18^O values of nearby rivers of −6.6‰ VSMOW (Rio Tzunutz) and −6.45 ‰ VSMOW (Rio Cahabón), which are typical for that location and altitude in Guatemala^64^ (Supplementary Fig. 1). We also completed three along-layer sampling transects (Supplementary Fig. 4); these data show no significant δ ^18^O increase away from the growth axis at depths of 0, 52.5, and 116 mm. These data suggest that kinetic isotope effects were not a controlling factor for the δ^18^O and δ^13^C values along individual growth layers. Combined, these data suggest that GU-RM1 was precipitated at or near isotopic equilibrium with regional annual precipitation. The damp stalagmite surface, high regional humidity, and apparent isotopic equilibrium between stalagmite calcite δ^18^O and nearby waters suggest that kinetic fractionation is not an important control on calcite δ^18^O.

### Climate model and idealized AMO simulations

The Max Planck Institute Earth system model for paleo-applications includes the atmospheric general circulation model ECHAM6 in its T63L47 configuration (corresponding to a longitudinal grid spacing of about 190 km at the tropics) and the ocean-sea ice model MPIOM in its GR15L40 configuration^65^. This climate model has been extensively studied regarding the characteristics of the AMO and its atmospheric teleconnections^35^ and its link with ocean dynamics in the equatorial Pacific^45^. When coupled, ECHAM6 is affected by a systematic bias in the partitioning of precipitation between land and sea in tropical regions compared to observations^66^. The idealized simulation employed here features 70-year long sinusoidal AMO oscillations that are imposed to the model via pattern nudging applied on upper-ocean potential temperature field in the North Atlantic basin^45^. Five consecutive AMO oscillations with a maximum amplitude of about 2 °C are simulated starting from a millennial control run. Further information about the nudging procedure and the simulation is provided in ref. ^45^. Due to the rather coarse model resolution and associated biases, we only focus on large-scale changes and avoid interpreting local features in Central America.

## Supplementary information

Supplementary Information

## Data Availability

The GU-RM1 data reported in this paper have been deposited in the cave section of the paleoclimatology data sets: https://www.ncdc.noaa.gov/paleo/study/28351
