# Supplementary material for: Initiation of a stable convective hydroclimatic regime in Central America circa 9000 years BP
Source: Nat Commun. 2020 Feb 5;11:716. doi: 10.1038/s41467-020-14490-y (PMC7002718; doi:10.1038/s41467-020-14490-y)
Supplement: Supplementary file 1 — Supplementary Information [file 41467_2020_14490_MOESM1_ESM.pdf]

Supplementary Information

Initiation of a stable convective hydroclimatic regime in Central America circa 9000 years BP

Winter et al.

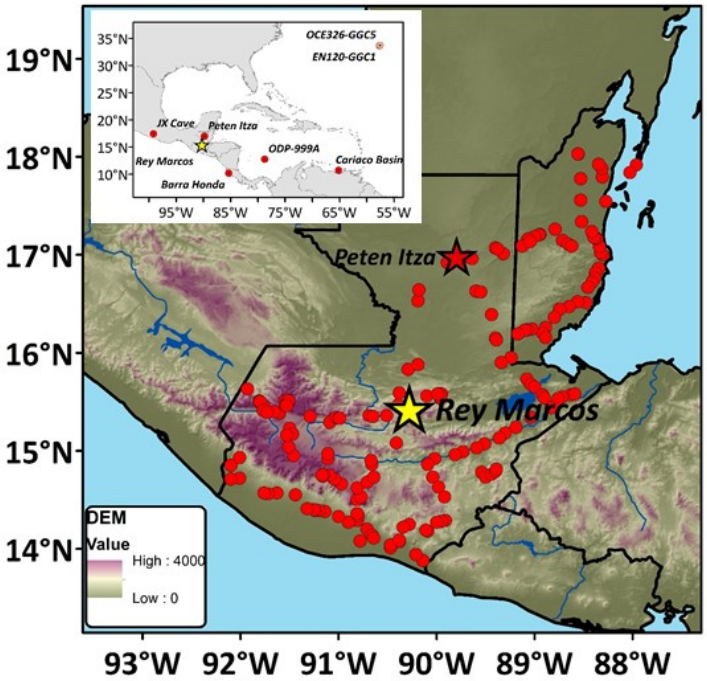

Supplementary Figure 1 – Location of Rey Marcos cave (marked by a yellow star). The location of surface water samples that provide isotopic baseline data for the interpretation of calcite-water equilibrium are shown in red circles, and Lago Peten Itzá is shown in a red star. Inset shows regional records in Figures 2 and 3; JX Cave indicates Juxtlahuaca Cave, Mexico; Barra Honda includes the records from Terciopelo Cave, Costa Rica. DEM stands for Digital Elevation Model (values in meters).

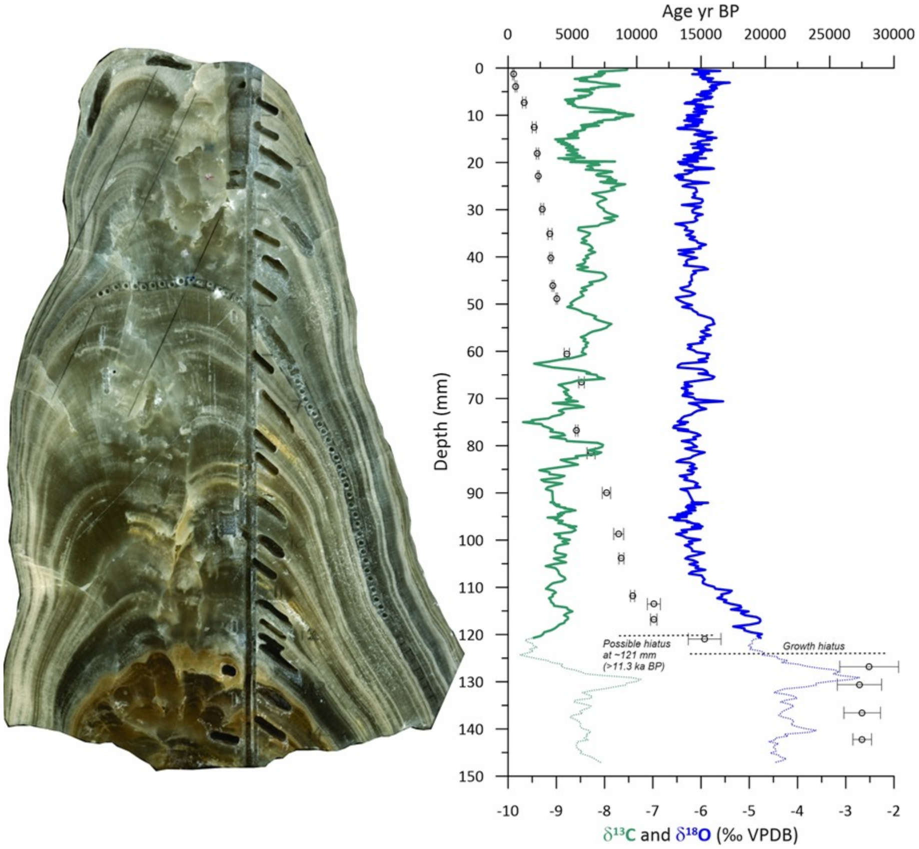

Supplementary Figure 2 – δ^{18}O, δ^{13}C, and age data against depth in GU- RM-1. The solid lines are the isotope data above 121 mm that were used in the paper and have robust U-series age control. The dotted thin lines are δ^{18}O data below 121 mm that were not used in the paper because of poor U-series age control. A likely hiatus at ~121 mm (horizontal dashed line) prevented establishment of a robust age model prior to the 11,320 ± 247 yr BP 230Th date. Another likely hiatus happened between the 15,279 ± 1273 and 27,315 ± 1722 yr BP dates. The pre-121 mm δ^{18}O data show that an even more extreme monsoon weakening happened prior to ~15,300 yr BP, but we are unable to resolve the chronology for this earlier section.

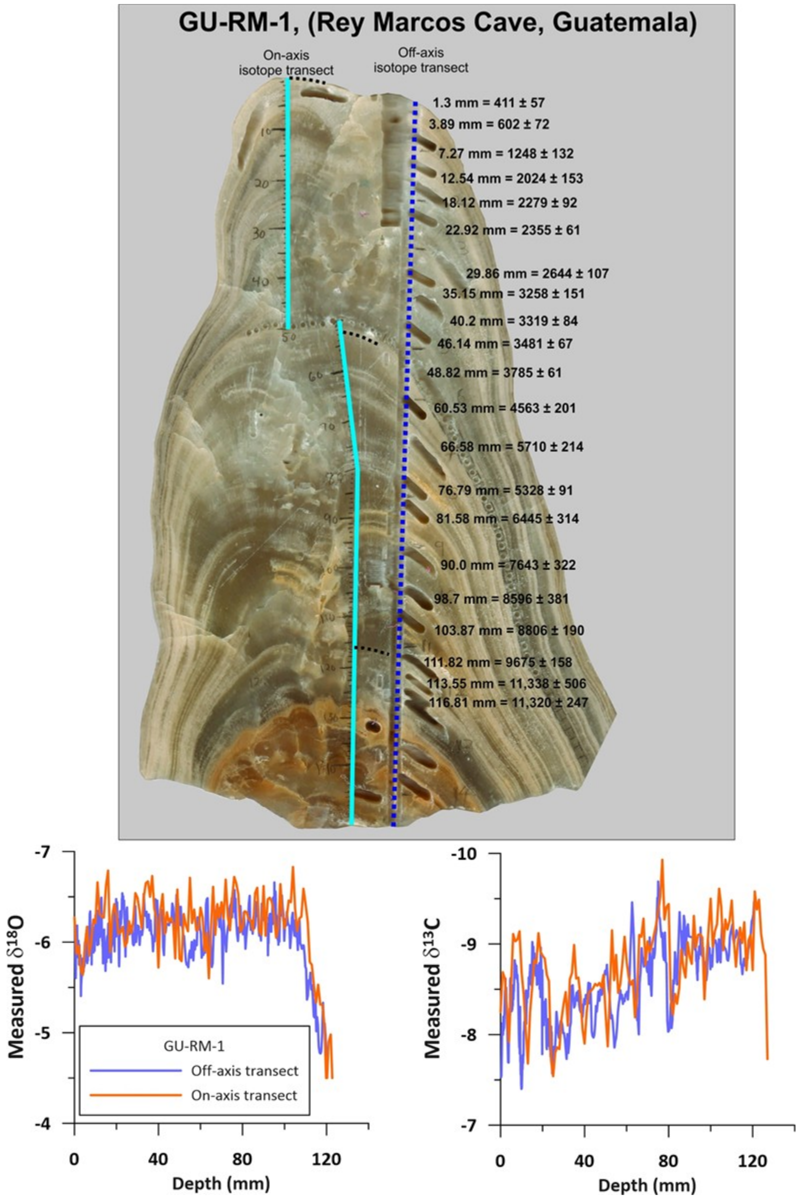

Supplementary Figure 3 – Comparison of off-axis and on-axis sampling transects. The off-axis milling transect is on the right (dotted blue line) with adjacent pits for U-series ages; the on-axis drilling path is shown (solid light blue line) on left. Along-layer isotope profiles are shown as dotted black lines at 0, 52.5, and 116 mm depths; data are shown in Supplementary Figure 4. The strong similarity of isotope profiles for the on- and off-axis transects show that no significant influence on the $\delta^{18}O$ and $\delta^{13}C$ profiles on the early Holocene rainfall strengthening.

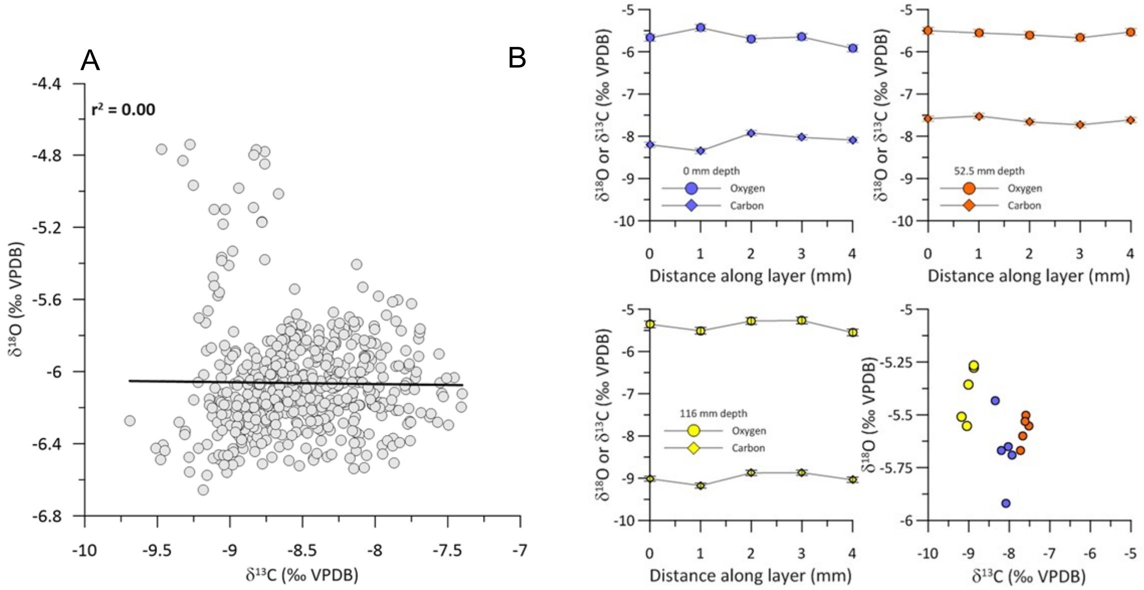

Supplementary Figure 4 –Test for kinetic fractionation. A) There is no relation between $\delta^{18}O$ – $\delta^{13}C$ indicating little kinetic alteration over the samples above 121 mm depth as used in this paper. The high $\delta^{18}O$ values are from the base of the stalagmite when convective activity was decreased in comparison to the post-9,000 yr BP wet conditions with lower $\delta^{18}O$ values. B) along-layer $\delta^{18}O$ – $\delta^{13}C$ analyses for three different depths (0, 52.5 and 116 mm) show no significant $\delta^{18}O$ increases. Locations of along-layer isotope transects are shown in Supplementary Figure 3. Together with evidence for equilibrium precipitation, these data show that the stalagmite has not experienced significant kinetic isotope effects.

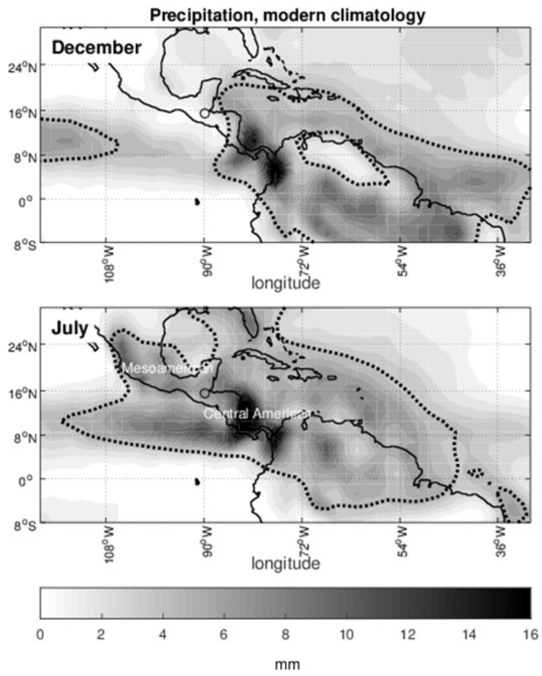

Supplementary Figure 5 – Modern (1948-2013 CE) precipitation climatology over Central America. Data are from NCAR reanalysis data for December (top) and July (bottom); The circle indicates the location of cave Rey Marcos where speleothem record GU-RM1 was collected. The dashed contour represents the 5 mm/day level after bilinear smoothing of the data and approximately corresponds to the ITCZ location.

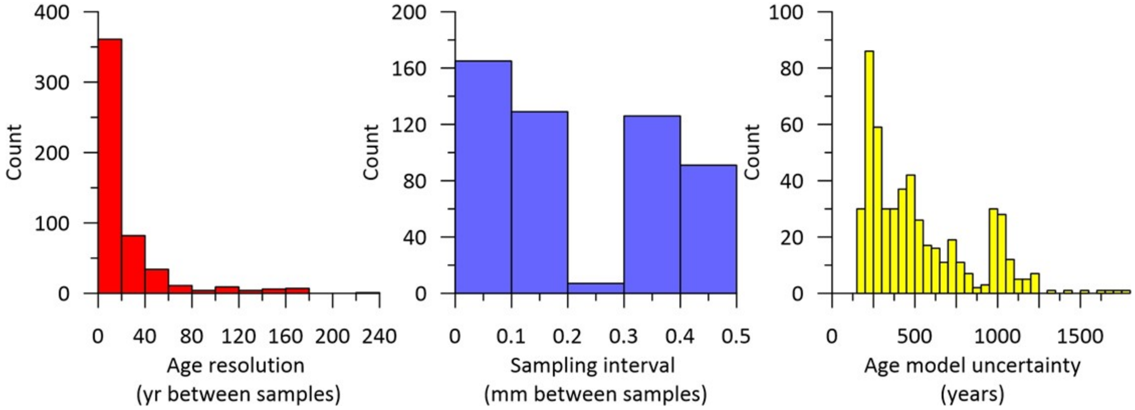

Supplementary Figure 6 – Histograms of age resolution, intervals, and age model uncertainties. Most of the subsampling was completed at resolution <0.4mm, and 83% of the samples have an age resolution of <40 years between samples (69% of samples <20 years). These data reveal that the main limitation is not sub-sampling interval but uncertainty on the chronology.

Supplementary Table 1

Supplementary Table 1. ^{230}Th dating results for stalagmite GU-RM-1. The age uncertainty is 2s.

Sample Number	Depth (mm)	^{238}U (ppb)	^{232}Th (ppt)	^{230}Th / ^{232}Th (atomic x10^{-6})	δ^{234}U* (measured)	^{230}Th / ^{238}U (activity)	^{230}Th Age (yr) (uncorrected)	^{230}Th Age (yr)** (corrected)	$\delta^{234}U_{Initial}$*** (corrected)	^{230}Th Age (yr BP)**** (corrected)	^{230}Th Age (yr CE) (corrected)
RM-1a-0-1	1.3	136 ±0	512 ±10	32 ±1	462.2 ±1.7	0.0074 ±0.0003	551 ±20	476 ±57	463 ±2	411 ±57	1539 ±57
RM-1a-1	3.9	135.3 ±0.2	610 ±12	37 ±2	459.1 ±1.6	0.0101 ±0.0004	755 ±33	665 ±72	460 ±2	602 ±72	1348 ±72
Date1-0-1	7.3	140.8 ±0.1	1278 ±26	36 ±1	469.1 ±1.5	0.0200 ±0.0005	1494 ±36	1314 ±132	471 ±1	1248 ±132	702 ±132
Date1-0-2	12.5	140.4 ±0.1	1415 ±28	50 ±2	482.2 ±1.7	0.0308 ±0.0008	2288 ±63	2090 ±153	485 ±2	2024 ±153	-74 ±153
RM-1a-1-1	18.1	137 ±0	885 ±18	85 ±2	489.4 ±1.6	0.0334 ±0.0003	2470 ±22	2344 ±92	493 ±2	2279 ±92	-329 ±92
Date1-2	22.9	151.6 ±0.1	522 ±11	161 ±4	485.4 ±2.1	0.0336 ±0.0005	2489 ±38	2421 ±61	489 ±2	2355 ±61	-405 ±61
RM-1a-2	29.9	121.4 ±0.2	876 ±18	88 ±2	486.5 ±2.1	0.0384 ±0.0005	2848 ±40	2707 ±107	490 ±2	2644 ±107	-694 ±107
Date2-0-1	35.2	155.8 ±0.2	1623 ±33	75 ±2	487.8 ±2.3	0.0474 ±0.0006	3527 ±47	3324 ±151	492 ±2	3258 ±151	-1308 ±151
RM-1a-2-1	40.2	154 ±0	911 ±18	131 ±3	486.5 ±1.9	0.0470 ±0.0003	3500 ±21	3384 ±84	491 ±2	3319 ±84	-1369 ±84
Date2-1-1	46.1	156.3 ±0.1	623 ±13	201 ±5	482.4 ±1.5	0.0486 ±0.0005	3625 ±38	3547 ±67	487 ±1	3481 ±67	-1531 ±67
RM-1a-3	48.8	151.7 ±0.2	581 ±12	226 ±5	483.5 ±2.1	0.0525 ±0.0004	3923 ±31	3848 ±61	489 ±2	3785 ±61	-1835 ±61
Date3-1	60.5	146.3 ±0.2	2050 ±41	77 ±2	483.2 ±1.8	0.0654 ±0.0007	4903 ±53	4629 ±201	490 ±2	4563 ±201	-2613 ±201
RM-1a-4*****	66.6	149.2 ±0.2	2270 ±46	87 ±2	480.0 ±1.7	0.0804 ±0.0005	6071 ±38	5773 ±214	488 ±2	5710 ±214	-3760 ±214
Date4-1	76.8	201.3 ±0.2	1241 ±25	196 ±4	484.5 ±1.6	0.0734 ±0.0004	5514 ±32	5394 ±91	492 ±2	5328 ±91	-3378 ±91
Date4-2	81.6	151.5 ±0.2	3417 ±69	68 ±1	492.3 ±1.9	0.0924 ±0.0007	6949 ±53	6511 ±314	501 ±2	6445 ±314	-4495 ±314
RM-1a-5	90.0	160.6 ±0.2	3728 ±75	77 ±2	489.2 ±1.9	0.1077 ±0.0005	8157 ±42	7706 ±322	500 ±2	7643 ±322	-5693 ±322
RM-1a-5-0-1	98.7	1114 ±2	30857 ±620	72 ±1	493.0 ±2.4	0.1213 ±0.0004	9198 ±37	8661 ±381	505 ±2	8596 ±381	-6646 ±381
Date5-1	103.9	173.0 ±0.2	2424 ±49	147 ±3	551.0 ±1.8	0.1252 ±0.0006	9133 ±43	8872 ±190	565 ±2	8806 ±190	-6856 ±190
RM-1a-6	111.8	137.6 ±0.2	1655 ±33	195 ±4	618.2 ±2.1	0.1419 ±0.0006	9952 ±45	9738 ±158	635 ±2	9675 ±158	-7725 ±158
GU-RM-6-0-1*****	113.6	131.6 ±0.1	5331 ±107	71 ±1	638.3 ±1.8	0.1734 ±0.0006	12117 ±47	11404 ±506	659 ±2	11338 ±506	-9388 ±506
GU-RM-6-0-2	116.8	80.1 ±0.1	1611 ±32	142 ±3	692.8 ±1.9	0.1738 ±0.0007	11728 ±50	11386 ±247	715 ±2	11320 ±247	-9370 ±247

U decay constants: $\lambda_{238} = 1.55125x10^{-10}$ (Jaffey et al., 1971) and $\lambda_{234} = 2.82206x10^{-6}$ (Cheng et al., 2013). Th decay constant: $\lambda_{230} = 9.1705x10^{-6}$ (Cheng et al., 2013).

*$\delta^{234}U = ([^{234}U/^{238}U]_{activity} - 1)x1000$. *** $\delta^{234}U_{initial}$ was calculated based on ^{230}Th age (T), i.e., $\delta^{234}U_{initial} = \delta^{234}U_{measured}$ x $e^{\lambda 234xT}$.

Corrected ^{230}Th ages assume the initial $^{230}Th/^{232}Th$ atomic ratio of 4.4 ±2.2 x10^{-6}. Those are the values for a material at secular equilibrium, with the bulk earth $^{232}Th/^{238}U$ value of 3.8. The errors are arbitrarily assumed to be 50%.

** Age in years before date of chemical preparation

****B.P. stands for “Before Present” where the “Present” is defined as the year 1950 A.D.

*****Excluded from age model
